# Deskilling dilemma: brain over automation

**DOI:** 10.3389/fmed.2026.1765692

**Published:** 2026-02-03

**Authors:** Shereen El Tarhouny, Amira Farghaly

**Affiliations:** 1 Health Profession Education Center, Ibn Sina National College for Medical Studies, Jeddah, Saudi Arabia; 2 Medical Biochemistry Department, Faculty of Medicine, Zagazig University, Zagazig, Egypt; 3 Department of Basic Medical Sciences, College of Medicine, Prince Sattam Bin Abdulaziz University, Al-Kharj, Saudi Arabia; 4 Medical Education Department, Faculty of Medicine, Suez Canal University, Ismailia, Egypt

**Keywords:** artificial intelligence, deskilling, medical education, over-automation, overreliance

## Introduction

Deskilling refers to the reduction of the skill level required to perform a task. In modern medical education and practice, it refers to the gradual erosion of independent clinical reasoning skills, together with crucial elements of clinical competence (1). Nowadays, this phenomenon represents a pattern of dependence on technology, especially Artificial Intelligence (AI), where tools and standardized approaches are designed to enhance efficiency and accuracy. Nevertheless, overreliance on technology can progressively displace essential skills across the full continuum of medical education, from the teaching and learning of medical students, through residency training, to everyday clinical practice in healthcare delivery (2).

In this opinion, we focus on early-career clinicians and residents, who are still in the phase of learning and consolidating clinical skills and may therefore be particularly vulnerable to AI-induced deskilling.

## AI in healthcare

AI has significant potential in medicine, alongside substantial challenges. AI applications are increasingly used for medical documentation, image interpretation, and analysis of large clinical and research datasets, offering improved efficiency and novel insights. Additionally, AI supports outbreak detection, disease identification through integrated clinical and genetic data, and hospital operations. Unlike other sectors, AI in healthcare requires rigorous testing, ethical oversight, and regulatory governance comparable to other medical technologies. AI and machine learning are viewed as tools that enhance, not replace, health professionals. By improving processes and decision-making, AI can free clinicians' time for meaningful patient interactions, ultimately contributing to higher-quality care and transforming medical practice (3).

Recent advances in AI are challenging the traditionally exclusive human role in diagnosis. AI may independently extract and synthesize information from records to support diagnosis. Although AI evolves rapidly and may be embedded invisibly within decision support systems, education on its impact is imperative. AI should be viewed as augmenting clinical reasoning, improving diagnostic accuracy, supporting triage, enhancing training, and freeing clinicians' time for more complex tasks, rather than replacing clinicians' clinical reasoning (4).

## The nature of deskilling

Contemporary clinical practice increasingly requires transferring knowledge to unfamiliar and ambiguous contexts, adopting emerging approaches, and operating effectively within the uncertainty of medicine. These competencies are labelled around the concept of adaptive expertise, which is crucial for ensuring safe, effective, and responsive clinical care. Deskilling represents a significant risk to adaptive expertise by encouraging surface learning, reducing problem-solving skills, failing to develop the habits and mindsets necessary for lifelong learning and professional identity formation (5). When the learning opportunities that develop adaptive expertise are systematically reduced, judgment, flexibility, and retention of mechanistic understanding weaken. The risk would be in producing clinicians who excel only in tightly defined, well-supported situations but struggle when faced with ambiguity or novel challenges. The risk of deskilling in the current generation of clinicians is considerably high with the surge of AI technologies that are available, abundant, and variable. The problem of deskilling will present on both professional and societal levels, with increasing ethical concerns about misdiagnosis (6).

## Neurological and psychological basis: offloading vicious cycle

When individuals repeatedly offload cognitive tasks to external support, neural adaptation occurs in ways that reduce independent learning and reasoning capacity. The prefrontal cortex becomes less active during clinical tasks, leading to diminished engagement in planning and problem-solving. The hippocampus, crucial for memory formation and retrieval, shows reduced involvement, resulting in weaker encoding and recall of clinical information. Dopaminergic reward systems reinforce the use of easy, externally supported strategies, making the brain increasingly likely to favor cognitive offloading over effortful thinking. Over time, these neurological changes shift cognitive processing from flexible, analytic networks to more automatic, habit-based circuits. This transformation makes individuals efficient in familiar, well-supported situations but less adaptable and resilient when facing novel or complex challenges (7).

## Cognitive deskilling

When learning environments provide explicit cues, frameworks, and predetermined pathways, the cognitive process of reasoning becomes more efficient. Learners perform competently when familiar prompts guide their thinking, but their performance weakens when those cues are absent, unfamiliar, or misleading. The ability to generate diagnostic hypotheses from ambiguous clinical data and to think through uncertainty rather than follow algorithms blindly diminishes with disuse. Skills, however, are not eliminated overnight but progressively displaced by pattern recognition and protocol adherence. Over time, this displacement produces clinicians who excel at matching presentations to known frameworks but struggle when patients deviate from textbook patterns.

## Diagnostic deskilling

Consolidation of clinical practice results from an iterative learning process. When this process is disrupted early on in clinical training, such as in undergraduate education or residency training, it leads to diminished exposure to common clinical presentations and, therefore, failure to develop pattern recognition and diagnostic fluency, skills that are highly needed for expert clinicians (7).

When clinicians become overly dependent on AI models, they rely less on their own skills and more on these models, assuming that they will always be more efficient and accurate, which may lead to better diagnosis (8). Overreliance on AI models will also lead to health practitioners being less confident in making independent decisions, potentially creating a cycle of dependence. AI tools may be perceived as rendering diagnostic processes less reliant on human judgment, thereby undermining their expertise and capacity to critically evaluate algorithmic outputs. This phenomenon is called diagnostic deskilling (5).

## Moral deskilling

Moral deskilling refers to the decline in ethical sensitivity and moral judgment resulting from over-reliance on technology. This diminished ethical capacity may leave clinicians less prepared to recognize when AI suggestions conflict with patients' best interests or values. The automation of clinical decision-making can distance clinicians from the moral dimensions of care, reducing their engagement with the ethical complexities inherent in medical practice. The non-technical, human-centric aspects of clinical care face similar erosion. Increasing focus on AI-generated results may lead clinicians to become preoccupied with interpreting algorithmic assessments rather than engaging in direct patient interaction. This shift threatens the ability to gather nuanced information through systematic history-taking and sensitively convey information to patients (8).

## Discussion

### Mitigation strategies against deskilling

AI implementation does not necessarily lead to deskilling of health practitioners; instead, it can reshape medical competencies and enhance clinical skill development. Clinicians' roles may shift toward supervising AI, validating outputs, and integrating recommendations into patient care. Additionally, AI can serve as an educational tool, supporting skill development and strengthening clinical judgment and diagnostic performance (5).

The aim of mitigating deskilling is not to reject AI technologies, but rather to ensure that they enhance clinical reasoning and lifelong learning capacities, instead of erasing them. Addressing cognitive deskilling requires intentional educational design early on in undergraduate and postgraduate medical education, to ensure the development of clinical reasoning skills through regular practice on unsupported reasoning and active problem-solving exercises (1). When it comes to assessment practices, there should be a balance between recognition and generation of information, with a focus on the assessment tasks that allow trainees to demonstrate problem-solving and clinical reasoning skills. Furthermore, more emphasis should be placed on assessing processes rather than products, by giving space to reflection exercises and closely monitoring the processes. To address the challenge of diagnostic deskilling, healthcare institutions should schedule practice sessions that are totally free from digital aids. In these sessions, trainees would be allowed to embrace certain amounts of inefficiency and risk, because challenge and complexity are integral parts of their learning process (9). Moral reskilling and upskilling should be encouraged in healthcare institutions by encouraging the practice of new moral skills and reinforcing learning by feedback and reflection exercises (10, 11).

Medical education curricula and training programs for residents should foster reflective exercises when trainees use AI technologies. These exercises will encourage trainees to explain their reasoning processes and critically reflect on how external aids influenced their thinking. Reframing educational objectives to train clinicians who can provide high-quality care both with and without AI and who can critically evaluate and verify AI outputs is essential. Faculty development programs are also required to equip educators with the skills to supervise students effectively, promote ethical and balanced use of AI, and identify early signs of deskilling while implementing appropriate mitigation strategies.

Teaching explicitly about the cognitive risks of overreliance on technological supports is of utmost importance, as it will foster awareness of when and how to use AI technologies productively. This can help trainees make better use of AI technologies. Healthcare institutions should have clear institutional policies for AI applications in training and in practice. This proactive approach would protect human expertise and make sure that adoption of AI technologies enhances rather than replace human expertise (7).

Drawing on emerging work on hybrid human–AI intelligence, AI systems can be intentionally designed to augment, rather than automate, clinical reasoning by prompting learners to articulate rationales, engage in contextual interpretation, and reflect on decision-making processes. When aligned with competency-based frameworks, AI-supported tools can be utilized to structure deliberate practice, provide adaptive feedback, and facilitate reflective supervision, all while preserving essential clinical skills. Importantly, this design orientation shifts the focus from efficiency-driven automation, which risks cognitive deskilling, to learning-oriented augmentation, where uncertainty, judgment, and sense-making remain central to professional formation. Such an approach underscores that the educational risk lies not in AI itself, but in how it is embedded within curricula, assessment practices, and supervision structures.

### Emerging challenges

Continuous over-reliance on AI and decision-support tools for learning and clinical reasoning, without reflection or critical appraisal of their output, may lead to deskilling and, more concerning, *never-skilling* (the failure to develop essential skills in the first place). Moreover, trainees are often more knowledgeable about technology than faculty, creating situations in which faculty may be expected to teach or supervise the use of tools with which they themselves are not fully competent. This mismatch can further complicate the learning process, affecting assessment, supervision, and even role modeling.

Ethical and legal concerns related to AI include patient privacy, data quality, algorithmic bias, and the risk of over-reliance by clinicians (12, 13). Evidence from high-stakes specialties such as neurosurgery highlights the risk that excessive dependence on AI could impede the acquisition and mastery of essential clinical skills, although a complementary human–AI skill synergy remains possible if carefully managed (12, 14). Beyond individual practitioners, AI-induced deskilling may have broader societal and organizational consequences, including increased risks of misdiagnosis, vulnerability to technological failures, and erosion of collective expertise, teamwork, and training capacity within healthcare institutions (15).

## Limitations and future direction

There is limited systematic evidence on AI-related deskilling in healthcare, including its timing, mechanisms, and affected groups. Its subtle nature and impact on hard-to-measure skills complicate assessment. Overall, evidence remains fragmented, and the field lacks a shared framework and coherent research agenda (5). This opinion provides insight into the nature of deskilling and potential strategies for its mitigation; however, further research is needed to examine the occurrence of deskilling among medical students and healthcare practitioners, as well as its impact on healthcare delivery and the practice of medicine.

AI should therefore be adopted only when it enhances outcomes without compromising the relational dimensions of care. It also underscores the importance of early interdisciplinary collaboration. The development of AI-enabled tools must be guided by clear conceptual and ethical understanding of the clinical tasks being automated, requiring input from data scientists, clinicians, ethicists, and patients (16).

## Conclusion

Deskilling represents a considerable threat to medical education and healthcare practice. Basically, it can lead to dependence on technology in diagnosing and managing patients, which might lead to a recession of the clinical competencies and deteriorate the quality of provided healthcare services. To prevent deskilling, medical schools and healthcare facilities should be vigilant about this impending threat and take preventive measures by applying changes to the design of teaching and learning activities, as well as assessment tasks, and institutional policies.
